# IL-27 Improves Prophylactic Protection Provided by a Dead Tumor Cell Vaccine in a Mouse Melanoma Model

**DOI:** 10.3389/fimmu.2022.884827

**Published:** 2022-04-21

**Authors:** Kyle Seaver, Olena Kourko, Katrina Gee, Peter A. Greer, Sameh Basta

**Affiliations:** ^1^ Department of Biomedical and Molecular Sciences, Queen’s University, Kingston, ON, Canada; ^2^ Department of Pathology and Molecular Medicine, Queen’s University, Kingston, ON, Canada

**Keywords:** IL-27, adjuvant, cancer vaccine, cell death, prophylactic, melanoma

## Abstract

The protocol used to induce cell death for generating vaccines from whole tumor cells is a critical consideration that impacts vaccine efficacy. Here we compared how different protocols used to induce cell death impacted protection provided by a prophylactic whole tumor cell vaccine in a mouse melanoma model. We found that melanoma cells exposed to γ-irradiation or lysis combined with UV-irradiation (LyUV) provided better protection against tumor challenge than lysis only or cells exposed to UV-irradiation. Furthermore, we found that the immunoregulatory cytokine, IL-27 enhanced protection against tumor growth in a dose-dependent manner when combined with either LyUV or γ-irradiated whole tumor cell vaccine preparations. Taken together, this data supports the use of LyUV as a potential protocol for developing whole tumor cell prophylactic cancer vaccines. We also showed that IL-27 can be used at low doses as a potent adjuvant in combination with LyUV or γ-irradiation treated cancer cells to improve the protection provided by a prophylactic cancer vaccine in a mouse melanoma model.

## Introduction

A key objective of cancer immunotherapy is to enhance immune recognition of tumors for elimination. Cancer vaccines can be delivered therapeutically to treat established tumors (1) or applied prophylactically to prevent tumor development or recurrence (2, 3). The immune system recognizes tumors using tumor associated antigens (TAAs), which provide targets for antigen specific CD8^+^ T lymphocyte (CTL) activation (4–8). Therefore, a successful cancer vaccine has the potential to promote robust CTL activation against TAAs (9). In addition to activating CTLs, cancer vaccines have also investigated the role of CD4^+^ T helper cells. Studies have elucidated a prominent role of CD4^+^ T cells in cancer vaccine clinical trials against multiple cancer types, including melanoma (10, 11).

In addition to providing TAAs, therapeutic cancer vaccines need to overcome an immunosuppressive tumor microenvironment (TME). Therefore, early intervention with prophylactic vaccination may be highly effective in preventing a tumor while also reducing the potential for recurrence following surgical removal of the tumor (12). Whole tumor cell vaccines consisting of dead tumor cells (DTCVs) can be used in prophylactic settings. Different protocols are used to produce DTCVs, including exposure to irradiation, γ-irradiation and ultraviolet (UV)-irradiation, oxidizing treatment, and lysis using heat-shock/snap-freezing and thawing (F/T) (13–15). Cancer cells exposed to these treatments can undergo immunogenic cell death (ICD) (16), releasing danger-associated molecular patterns (DAMPs), such as adenosine triphosphate (ATP), high mobility group box protein 1 (HMGB1), and heat shock proteins (HSPs) (17). DAMPs can activate professional antigen presenting cells (pAPCs), including dendritic cells (DCs). This activation is characterized by an increase in co-stimulatory molecule expression and release of cytokines that can enhance T cell activation (18, 19). CTL activation can be achieved when pAPCs present TAAs on major histocompatibility complex (MHC) class I to CTLs *via* antigen cross-presentation (20–23). DTCVs reduce the need to select a predictive TAA as it provides access to all potential tumor antigens, including uncharacterized and unique antigens not yet identified. Additionally, cancer cell variants can escape immune detection by downregulating antigens. Using DTCVs in a prophylactic setting may reduce escape variants from arising because they provide access to multiple antigens at once (24). However, tumor cells alone are poorly immunogenic, and studies have investigated how adjuvants can improve cancer vaccine efficacy (16).

The use of cytokines as adjuvants to enhance cancer vaccine efficacy has been well documented and can influence both innate and adaptive immune responses (25–28). Interleukin (IL)-12, for example, has been used as a cancer vaccine adjuvant; however, toxicity is a concern, and dosage requires careful consideration (29, 30). IL-27, a cytokine belonging to the IL-12 family of cytokines, has been identified as a potential cancer vaccine adjuvant (31). IL-27 can signal in T cells, macrophages, and monocytes while also directly impacting cancer cell death and proliferation (32, 33). Although IL-27 has been associated with both pro- and anti-tumor effects (34), elevated levels of IL-27 have demonstrated success in reducing cancer progression (31, 35). However, the use of IL-27 as an adjuvant to improve prophylactic cancer vaccines needs further investigation. Furthermore, understanding the impacts of different doses of IL-27 in combination with DTCVs has yet to be investigated.

In the present study, we examined how different protocols were used to generate a DTCV in combination with recombinant mouse (rm)IL-27 as an adjuvant, impact tumor growth focusing on the potential MHC-I/CD8^+^ T cell interactions. Using the B16-OVA murine melanoma model, we determined that the addition of rmIL-27 at a lower dose, rather than a higher dose, improved protection by a DTCV against tumor challenge. We also showed that despite the added protection against initial tumor challenge with the addition of rmIL-27 to a DTCV, rmIL-27 did not protect against tumor rechallenge. These results have implications on the potential use of IL-27 as an adjuvant in combination with vaccines generated from whole tumor cells.

## Methods

### Mice and Cell Lines

Male and female C57BL/6 (H-2^b^) mice (6-8 weeks old) were purchased from JAX^®^ Laboratories (Bar Harbour, USA) and kept under specific pathogen-free conditions. All animal experiments were conducted in accordance with the Canadian Council of Animal Use and approved by Queen’s University Animal Care Services.

The murine melanoma cell line B16F10 (H-2k^b^) was maintained in DMEM (Gibco, Fisher Scientific, Canada) supplemented with 5% FBS (Gibco, Fisher Scientific, Canada) (36). B16F10 cells transfected with chicken ovalbumin (OVA), a gift from Dr. Yewdell (NIAID, NIH, USA), were maintained under 500 μg/mL of G418 sulfate (Bioshop, Canada) selection in complete DMEM medium. The DC2.4 cell line (kindly provided by Dr. Rock, University of Massachusetts Medical School, USA), isolated from C57BL/6 mice bone marrow and transduced with retroviral vectors expressing murine granulocyte-macrophage colony-stimulating factor (GM-CSF) and myc and raf oncogenes (37), was maintained in RPMI media (Gibco, Fisher Scientific, Canada) supplemented with 5% FBS. All cells were maintained at 37°C and 5% CO_2_.

### Induction of Cell Death

B16-OVA cells were exposed to one of the following protocols of inducing cell death. *Cell lysis*: five consecutive rounds of F/T where cells were frozen using liquid nitrogen and thawed in a water bath at 37°C. *Ultraviolet-irradiation (UV-irradiation)*: Cells were exposed to UV-irradiation at a total exposure of 1500 mJ//cm^2^ using a CL-1000 ultraviolet crosslinker (Ultra-Violet Products Ltd., United Kingdom). *LyUV*: Cells were exposed to a single F/T cycle followed by UV-irradiation, as previously described (38, 39). *γ-irradiation*: Cells were exposed to 60 Gys of irradiation using a cesium irradiator as the source (Cs^137^ GammaCell 20 Irradiator, Queen’s University).

### Microscopy

Light microscopy was used to visualize the morphology of cancer cells following the protocols used to induce cell death. Cells were seeded into 6-well plates at a density of 1x10^6^ cell/well and observed using a light microscope (Lecia DM IRE2, Germany) at 20X magnification. Images were acquired using Lecia DFC340 cooled monochrome digital camera.

### Detection of Cell Viability

To measure the induction of cell death, annexin-V (AV) and propidium iodide (PI) staining was conducted as outlined by the manufacturer. Briefly, B16-OVA cells were washed twice in AV binding buffer (10 mM Hepes, pH 7.4, 0.14mM NaCl and 2.5 mM CaCl_2_) followed by staining with APC-conjugated AV (Biolegend, USA) for 15 min protected from light at room temperature. PI (Biolegend, USA) was then added 5 min before acquisition at a concentration of 1.5 μg/mL. Data was acquired using a CytoFLEX flow cytometer (Beckman Coulter, USA) and analyzed using FlowJo software (BD, USA).

### Evaluation of MHC-I Expression on Irradiated Tumor Cell by Flow Cytometry

Following exposure to either γ-irradiation or UV-irradiation or treatment with 50 ng/mL of interferon-γ (IFN-γ) (Shenandoah Biotechnology, USA), B16-OVA cells were incubated for 18 hrs. in a 6-well plate at a density of 1.0x10^6^ cells/well. After incubation, the cells were harvested and washed with 1X PBS, then transferred to a 96-well round-bottom plate (Corning, USA). Cells were then washed twice in flow staining buffer (1X PBS, 0.1% sodium azide, 1% BSA) and then stained with PE-anti-MHC-I (Biolegend, clone: 28-8-6) for 30 min at 4°C. Data was acquired using a CytoFLEX flow cytometer and analyzed using FlowJo software (BD, USA).

### Co-Incubation of DC2.4 Cells With Dead B16-OVA Supernatants

B16-OVA cells were exposed to the indicated protocol of inducing cell death as described earlier. After exposure, cancer cells were left to incubate for 24 hrs. in complete RPMI media at a concentration of 1.0x10^6^ cells/mL in a 6-well plate (Corning, USA) constituting the tumor conditioned media (TCM). Following the incubation period, supernatants were collected and centrifuged at 1000g for 5 min to remove debris and immediately added at a 1:1 ratio by volume (TCM: complete media) to 1.0x10^6^ DC2.4 cells in a total volume of 1.5 mL. The DC2.4 cells were subsequently left to incubate for 24 hrs, after which cells were collected and prepared for flow cytometry, as previously described. Cells were then stained with the following antibodies: FITC-anti-MHC-II IA/IE (Biolegend, clone: M5/114.15.2), PE-anti-CD80 (Biolegend, clone: 16-10A1), APC-anti-CD86 (Biolegend, clone: GL-1), and PerCP-anti-CD40 (Biolegend, clone: 3/23) for 30 min at 4°C. Data was acquired using a CytoFLEX flow cytometer and analyzed using FlowJo software (BD, USA).

### Phagocytosis Assay

The phagocytosis assay was performed as previously reported (40). Briefly, cancer cells were stained with carboxyfluorescein succinimidyl ester (CFSE) (0.2 μM/mL) for 15 min at 37°C then washed extensively and exposed to the indicated protocols to induce cell death. Cancer cells were then co-cultured with DC2.4 cells at a ratio of 3:1 in a 96-well round-bottom plate for 3 hrs. at 37°C in a total volume of 200 μL/well. After the co-incubation, DCs were stained with PE-Cy7-anti-CD11c (Biolegend, clone: N418) for 30 min at 4°C. Data was acquired using a CytoFLEX flow cytometer and analyzed using FlowJo software to identify the cells that were CFSE^+^/CD11c^+^ double-positive cells.

### Preparation of a Prophylactic Cancer Vaccine Containing IL-27

B16-OVA cells were harvested and exposed to the indicated protocol to induce cell death. Cells were then resuspended at 5.0x10^6^ cells in 0.2 mL 1X PBS and delivered to each mouse *via* intraperitoneal (i.p) injection. Mice were injected with the corresponding vaccine 14 and 7 days before the tumor challenge. For vaccines that included rmIL-27 (Biolegend, USA), mice received 0.2 mL of either 10 ng/mouse or 100 ng/mouse of rmIL-27 suspended in 1X PBS alone or with dead tumor cells. For tumor engraftment, 1.0x10^6^ live B16-OVA cells were injected subcutaneously (s.c) into the right hind flank. Tumor growth was monitored by calipers every second day by measuring volume using the modified ellipsoidal formula: V=1/2 (length x widgth^2^) (41).

For tumor rechallenge experiments, mice that remained tumor free following the prophylactic vaccination and tumor engraftment were rechallenged on day 60 with 1.0x10^6^ live B16-OVA cells s.c into the opposite hind flank of the original engraftment.

### Statistical Analysis

Statistical significance was determined using GraphPad Prism. Comparison between two groups was done using Student’s t-test. One-way ANOVA was used when comparing differences between more than two groups. For Kaplan-Meier survival curves the Log-rank (Mantel-Cox) test was used. All values are reported as mean ± SD. A p-value ≤0.05 was considered significant.

## Results

### Cell Morphology Is Influenced by the Protocol Used to Induce Cell Death

The protocol used to induce cell death can result in differences in the level of protection observed by a prophylactic vaccine. Using B16-OVA melanoma cells, we visualized the impact of four different protocols used to induce cell death. Cancer cells were exposed to five consecutive rounds of freeze/thaw (lysis) which resulted in higher mean fluorescence intensity (MFI) of PI-positive cells although no differences in the percent of PI-positive cells was detected when compared to one round of lysis (**Supplementary Figure 1**). B16 cells were exposed to UV-irradiation at a total exposure of 1500 mJ/cm^2^, either delivered alone (UV-irradiation) or in combination with a single round of F/T (LyUV) as has been previously described (40, 42). For γ-irradiation, a range of doses (20 – 100 Gy) were tested but yielded no differences in the induction of cell death 24 hrs. after exposure (**Supplementary Figure 2**), or proliferation (data not shown). Based on these results and previous literature, we used 60 Gys of γ-irradiation exposure based on previous literature using B16 cells (43).

**Figure 1:**
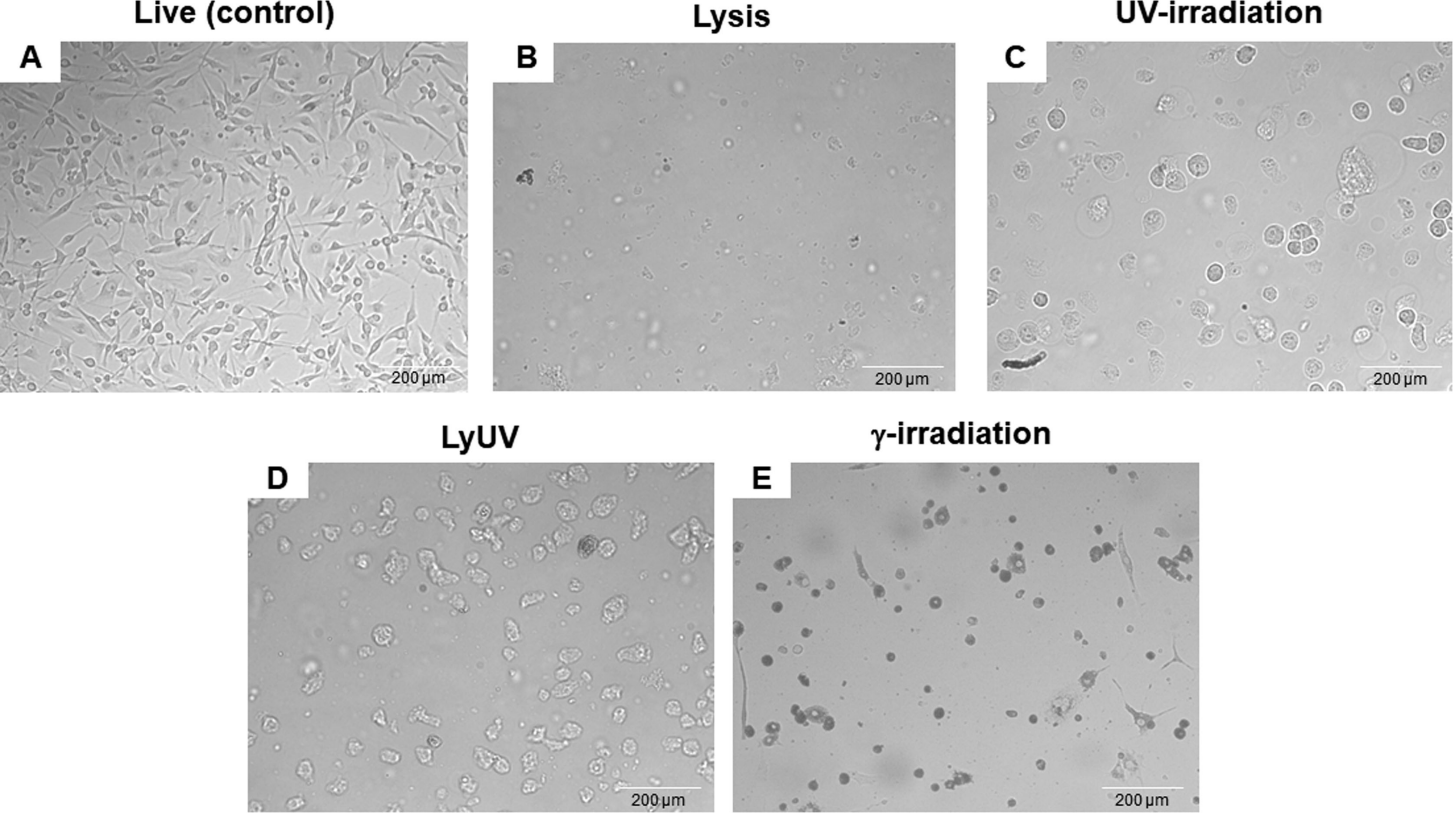
Differences in cell morphology 24 hrs. after induction of cell death. Cancer cells were exposed to lysis (5 cycles of F/T), UV-irradiation (1500 mJ/cm^2^), LyUV (1 round of F/T followed by 1500 mJ/cm^2^ UV-irradiation), or γ-irradiation (60 Gys). After exposure, cancer cells were seeded into a 6-well plate at 1.0x10^6^ cells/well and left to incubate for 25 hours. After the incubation period, morphological differences were observed by light microscopy at 20X magnification. **(A)** Live cancer cells. **(B)** Cells exposed to lysis. **(C)** UV-irradiated cells. **(D)** LyUV treated cells. **(E)** Cells exposed to γ-irradiation. One representative experiment of three is shown.

Following induction of cell death, B16-OVA cells were incubated for 24 hrs, and morphological differences and adherence were determined *via* light microscopy (**Figure 1**). Exposure to lysis resulted in few detectable intact cells with a large quantity of debris. Exposure to UV-irradiation did not yield adherent cells, most cells remained intact with a circular and swelled appearance compared to the elongated appearance of live (control) B16-OVA cells. Interestingly, LyUV yielded a combination of debris (similar to lysis) and intact cells (similar to UV). Compared to UV-irradiation, cells exposed to γ-irradiation did not demonstrate the same swelled appearance and yielded a mixture of adherent and non-adherent cells. Taken together, each protocol used to induce cell death yields morphological differences.

### Generating Apoptotic and Necrotic Cancer Cells for Use in a DTCV

**Figure 2:**
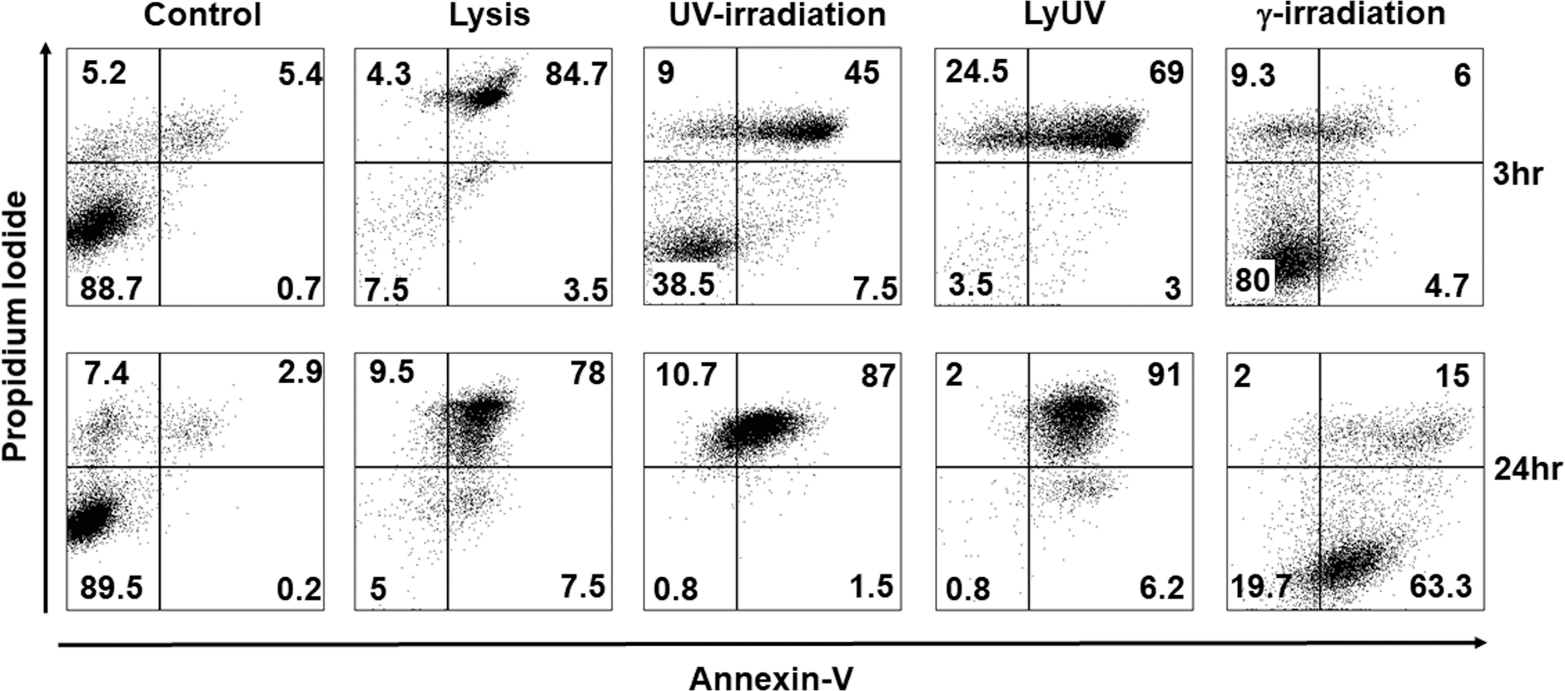
Comparison of cancer cell death in protocols used for DTCV production. Cancer cells were exposed to the indicated method of cell death and left to incubate for either 3 hrs. (top panels) or 24 hrs. (bottom panels). Cells were then examined by flow cytometry for annexin V and propidium iodide (AV/PI) staining. Percentages of cells found in each quadrant are the average from 4 independent experiments. Representative dot plots are depicted.

With observable differences in cell morphology, we next assessed cell viability using AV and PI staining at 3 hrs. and 24 hrs. after cell death was induced. After 3 hrs. of incubation following induction of cell death, lysis and LyUV resulted in a majority of the cells being late apoptotic (AV^+^/PI^+^) with 84.7% and 69%, respectively, with LyUV yielding a greater number of necrotic cells (AV^-^/PI^+^, 24.5%) (**Figure 2**). Cell viability following exposure to lysis and LyUV remained similar between the 3 hr and 24 hr time points. There were differences observed in cell viability when B16-OVA cells were exposed to UV- or γ-irradiation. Following 3 hrs. post-exposure to UV- or γ-irradiation, cancer cells that remained alive (AV^-^/PI^-^) were 38.5% and 80%, respectively (**Figure 2**). After 24 hrs. post-exposure, UV-irradiation resulted in most of the cells being late apoptotic (87%) whereas γ-irradiation resulted in the majority of cells being early apoptotic (AV^+^/PI^-^) (63.3%) (**Figure 2**). Overall, these results indicated that lysis and LyUV are comparable in the type of cell death induced, while UV- and γ-irradiation result in different proportions of apoptotic and necrotic cells while requiring 24 hrs. to yield the greatest reduction in cell viability.

### Irradiation Can Promote B16-OVA Cell Immunogenicity by Increasing MHC-I Expression

**Figure 3:**
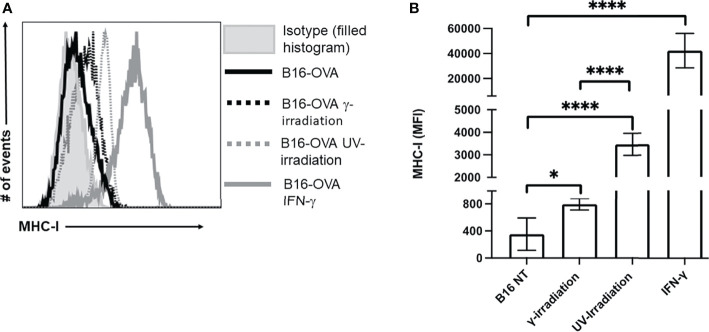
Exposure to UV- or γ-irradiation enhanced expression of MHC-I on B16-OVA cells. B16-OVA cancer cells were exposed to either 1500 mJ/cm^2^ of UV-irradiation, 60 Gys of γ-irradiation, 50 ng/mL of IFN-γ, or left untreated. The cancer cells were then left to incubate for 18hrs. followed by staining for MHC-I and analysis by flow cytometry. **(A)** MHC-I surface expression on B16-OVA cells. The histogram shown is one representative experiment of 5 independent experiments. The filled histogram represents the isotype control, the solid black line is untreated B16-OVA cells (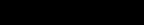), the dotted black line is B16-OVA cells exposed to γ-irradiation (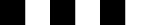), dotted grey line is B16-OVA cell exposed to UV-irradiation (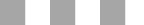), and the solid grey line is B16-OVA cell treated with IFN-γ (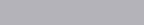). **(B)** Bar graph showing the mean fluorescence intensity (MFI) ± SD of 5 independent experiments. *p < 0.05,****p ≤ 0.0001.

In addition to inducing cell death, exposure to irradiation can enhance MHC-I expression (44–47). Increasing MHC-I expression has the potential to enhance CTL recognition of poorly immunogenic tumor cells. The expression of MHC-I on B16-OVA was measured by flow cytometry, and IFN-γ (50 ng/mL) stimulation was used as a positive control. We observed a significant increase in MHC-I on B16-OVA cells exposed to either UV- or γ-irradiation (**Figure 3A**). Compared to γ-irradiation, UV-irradiation resulted in a greater increase of MHC-I expression, although neither form of irradiation could increase the expression of MHC-I to the same extent as IFN-γ (**Figure 3B**). These results indicate that both forms of irradiation increase the expression of MHC-I on B16-OVA cancer cells, although UV-irradiation does this to a greater extent.

### Supernatants From B16-OVA Cells Impact Dendritic Cell Activation Which Is Dependent on the Protocol Used to Induce Cell Death

**Figure 4:**
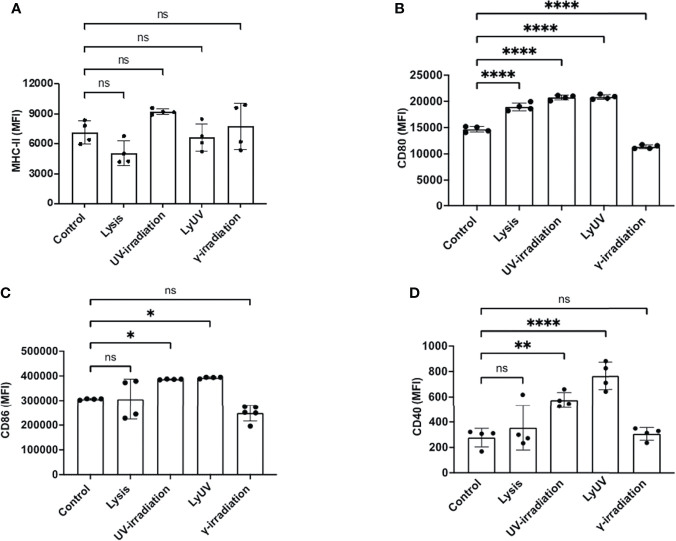
Supernatants from dead tumour cells induce different expression patterns of co-stimulatory molecules on dendritic cells. After induction of cell death, cancer cells were seeded into a 12-well plate at a final concentration of 1.0x10^6^/mL and left to incubate for 24 hrs. After which supernatants were collected and spun down at 1000g for 5 min to remove cell debris. The tumour cell-conditioned media (TCM) was then added to the DCs at a ratio of 1:1 (TCM: complete media). The DCs were incubated for 24 hrs. in the presence or absence of TCM before being analyzed by flow cytometry for surface marker expression. Bar graphs show expression levels of **(A)** MHC-II, **(B)** CD80, **(C)** CD86, and **(D)** CD40. The control represents DCs incubated in complete media alone. Each bar graph shows the mean fluorescence intensity (MFI) ± SD of 4 independent experiments. *p < 0.05,**p < 0.01, ****p ≤  0.0001. ns denotes not significant.

In addition to inducing cell death, lysis and irradiation can result in the release of DAMPs, which can influence DC activation, assessed by an increased expression of co-stimulatory molecules (CD80, CD86, CD40) and MHC-II (48). To evaluate DC activation, we used the well-characterized dendritic cell line, DC2.4, originally derived from the bone marrow of C57BL/6 mice (37). Regardless of the protocol used to induce cell death, B16-OVA supernatants did not affect the expression of MHC-II on DC2.4 cells (**Figure 4A**). DC2.4 cultured in lysis, LyUV or UV-irradiation supernatants had a significant increase in CD80, whereas a significant reduction in CD80 was seen in DC2.4 cell culture in γ-irradiation supernatants (**Figure 4B**). DC2.4 cells incubated with LyUV or UV-irradiation supernatants both displayed an increase in CD86 and CD40 expression (**Figures 4C, D**). Taken together, these results indicate that the protocol used to induce cell death can influence the impact of the TCM on DC2.4 activation.

### The Mode of Cancer Cell Death Influences the Rate of Their Phagocytosis by Dendritic Cells

**Figure 5:**
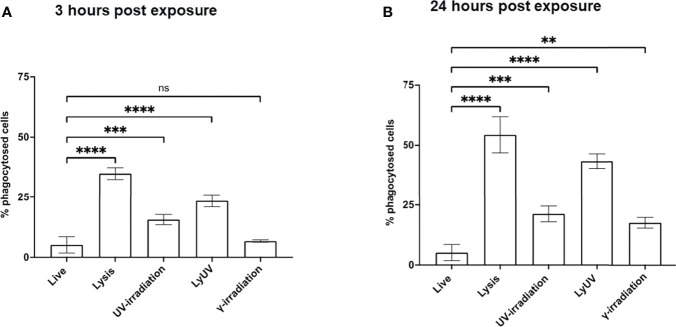
Phagocytosis of B16-OVA cells by dendritic cells is influenced by the cell death protocol employed. Cancer cells were stained with 0.2 μM of CFSE for 15 min at room temperature before induction of cell death. After which cancer cells were left to rest for either **(A)** 3 hrs. or **(B)** 24 hrs., then co-incubated with dendritic cells for 3 hrs. at a 3:1 ratio (cancer cells: DC). Cancer cell phagocytosis was determined by flow cytometry and identified by double-positive cells (CFSE^+^/CD11c^+^). Bar graphs show percent phagocytosis as ± SD of 3 independent experiments. **p < 0.01, ***p < 0.001 ****p ≤ 0.0001. ns denotes not significant.

Phagocytosis of dead cancer cells by antigen presenting cells leads to TAA presentation resulting in T cell priming. Therefore, we next determined how DCs phagocytosed tumor cells following exposure to each of the methods of cell death being evaluated. Cancer cells were stained with CFSE and exposed to the protocols of cell death induction. After exposure cancer cells were left to rest for 3- or 24-hrs, after which the cancer cells were co-incubated with DCs (3:1) for 3 hrs. to evaluate phagocytosis. Following co-incubation, cancer cell phagocytosis by DCs was determined based on the percent of double-positive DC cells (CFSE^+^/CD11c^+^). After incubation times of 3 and 24 hrs, we observed the greatest amount of phagocytosis with lysed cells (**Figure 5**). 25% of cancer cells exposed to LyUV were phagocytosed after 3 hrs, which increased to 40% if the cells were left to incubate for 24 hrs. before incubation with DCs. In comparison, UV- and γ-irradiation resulted in lower percentages of phagocytosis after 3 hrs., 15% and 7% respectively. However, if the cancer cells were left for 24 hrs. before co-incubation with DCs, the rate of phagocytosis increased significantly compared to live cells with UV-irradiation increasing to 21% and γ-irradiation increasing to 17%. Overall, these results indicate that when cancer cells are exposed to either lysis or LyUV, phagocytosis by DCs can occur more rapidly than when cancer cells are exposed to UV- or γ-irradiation.

### Prophylactic Vaccination With LyUV-Treated or γ-Irradiated B16-OVA Cells Promotes Better Tumor Free Survival When Compared to Unvaccinated Control Mice

**Figure 6:**
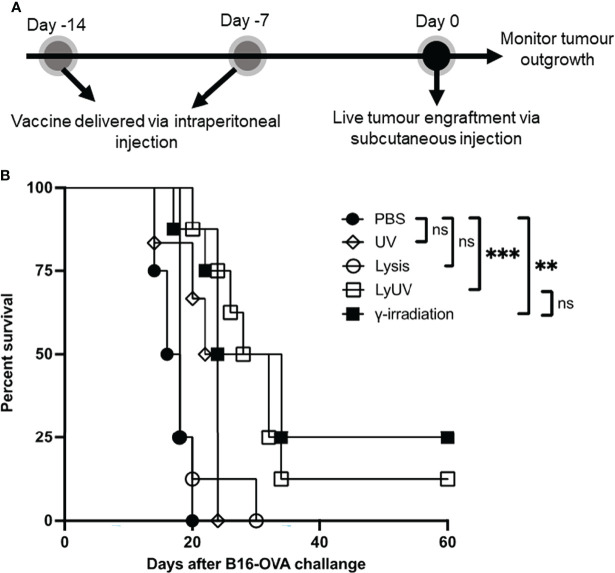
A prophylactic cancer vaccine consisting of dead tumour cells following LyUV or γ-irradiation can enhance tumour free survival. **(A)** Schematic representation of prophylactic vaccination preparation. A total of 5.0x10^6^ B16-OVA cells were exposed to the indicated protocols of inducing cells death (lysis n = 8, UV n = 6, LyUV n = 8 or γ-irradiation n = 8) and injected i.p. into C57BL/6 mice 14 and 7 days before live tumour engraftment. Control mice were injected with 1X PBS (n = 4) instead of tumour cells for each vaccination. Seven days after the second vaccination (day 0) mice were injected with 1.0x10^6^ live B16-OVA cells subcutaneously and tumour growth was then monitored every second day for the duration of the experiment. **(B)** Kaplan-Meier survival curve of mice receiving each of the vaccinations. **p < 0.005, ***p < 0.001. ns denotes not significant.

We next proceeded to determine how each protocol used to induce cell death impacted the efficacy of a DTCV delivered in a prophylactic model. A prime-boost model of vaccination showed that two DTCV injections provided greater tumor protection than a single injection (**Supplementary Figure 3**). Vaccines were administered immediately following induction of cell death on days -14 and -7, followed by live tumor challenge on day 0 (**Figure 6A**).

Compared to PBS control mice, prophylactic vaccines consisting of lysed or UV-irradiated B16-OVA cancer cells did not provide significant protection against tumor challenge (**Figure 6B**). However, increased survival was observed when mice were vaccinated with B16-OVA cells exposed to LyUV or γ-irradiation, with 12% and 25% of the mice remaining tumor free for 60 days post tumor engraftment, respectively (**Figure 6B**). These results indicate that in addition to γ-irradiation, LyUV has the potential to be used as a protocol for generating DTCV and should be explored further in cancer vaccine development.

### IL-27 Improves the Efficacy of the Prophylactic Cancer Vaccine With a Lower Dose Providing Enhanced Protection

**Figure 7:**
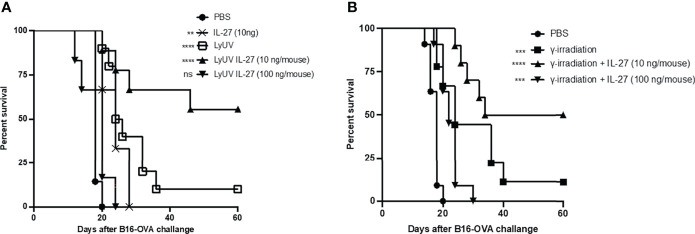
The addition of IL-27 to the DCTV at 10 ng/mouse enhances protection against tumour progression. **(A)** Cancer cells death was induced by LyUV, whereby cancer cells were exposed to a single round of F/T followed by 1500 mJ/cm^2^ of UV-irradiation. A total of 5.0x10^6^ LyUV treated cells in the presence or absence of 10 ng/mouse or 100 ng/mouse of IL-27 were injected i.p. into C57BL/6 mice. Vaccines were delivered 14 and 7 days before live tumour engraftment. Control mice were injected with 1X PBS instead of tumour cells for each vaccination. Seven days after the second vaccination (day 0) mice were injected with 1.0x10^6^ live B16-OVA cells subcutaneously and tumour growth was then monitored. **(B)** Following the same vaccination schedule as in A, however, cancer cells death was induced by γ-irradiation at a dose of 60 Gys. Kapan-Meier survival graph of n= 7 mice. **p < 0.01, ***p < 0.001, ****p < 0.0001. ns denotes not significant. The statistical difference is compared to PBS for each vaccination group.

In our model, we observed partial protection against tumor growth using B16-OVA cells that were exposed to LyUV or γ-irradiation. Therefore, we wanted to determine if rmIL-27 could influence the efficacy of the LyUV or γ-irradiated DTCV. Using the same vaccination schedule as previously described (**Figure 6A**), rmIL-27 was added at 10 or 100 ng/mouse in combination with the DTCV (LyUV or γ-irradiation) at days -14 and -7. While rmIL-27 alone at 10 ng/mouse showed a slight increase in protection relative to PBS, 100 ng/mouse rmIL-27 did not (data not shown). The combination of rmIL-27 at 10 ng/mouse provided significant improvement in protection in both LyUV and γ-irradiation DTCV models compared to unvaccinated controls (PBS), with ≥50% of the mice remaining tumor free at 60 days post engraftment (**Figure 7**). Interestingly, the combination of rmIL-27 at 100 ng/mouse to mice vaccinated with either LyUV or γ-irradiation based DTCV did not improve protection when compared to the DTCV alone. We also observed a similar trend with DTCVs produced using the B16 melanoma cells that do not express OVA following LyUV exposure, where the lower dose of rmIL-27 provided better protection than the higher dose (**Supplementary Figure 4**). Although the use of rmIL-27 as an adjuvant in our model provided significant protection against tumor growth, the differential effects observed with the addition of rmIL-27 indicate that the dose required careful consideration.

### IL-27 Can Improve Protection Against Initial Tumor Engraftment but Not Tumor Rechallenge

**Figure 8:**
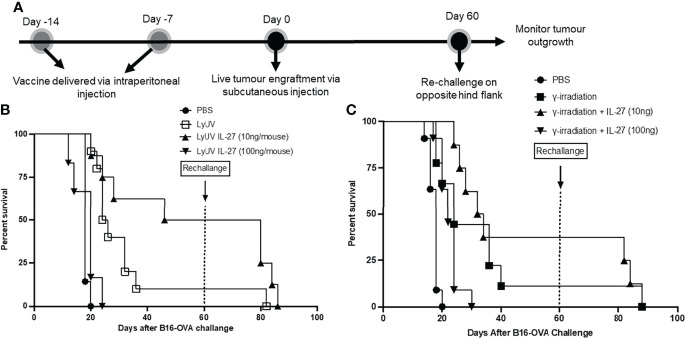
The use of IL-27 as a vaccine adjuvant does not provide long-term protection against tumour rechallenge. **(A)** Schematic representation of prophylactic vaccination preparation with tumour rechallenge. A total of 5.0x10^6^ B16-OVA cells were exposed to the indicated protocol of inducing cells death (LyUV or γ-irradiation) in the presence or absence of 10 ng/mouse or 100 ng/mouse of rmIL-27 and subsequently injected i.p. into C57BL/6 mice 14 and 7 days before live B16-OVA cancer cell engraftment. Control mice were injected with 1X PBS instead of cancer cells for each vaccination. Seven days after the second vaccination (day 0) mice were injected with 1.0x10^6^ live B16-OVA cells subcutaneously and tumour growth was then monitored. Mice that remained tumour free were rechallenged on day 60 *via* subcutaneous injection with 1.0x10^6^ live B16-OVA cells over the opposite hind flank. **(B, C)** LyUV and γ-irradiation Kapan-Meier survival curve. No significant differences were observed.

With the increased protection observed using rmIL-27 as a vaccine adjuvant, we next evaluated whether the addition of rmIL-27 could provide long-term protection in a tumor rechallenge model. To assess this, mice that remained tumor free after the first challenge were later rechallenged at day 60 (**Figure 8A**). Upon rechallenge, all mice that were clear of tumors initially after 60 days exhibited rapid tumor growth which resulted in no significant improvement in tumor free survival when compared to the age-matched controls challenged at day 60 (**Figures 8B, C**). This indicates that in our model rmIL-27, as an adjuvant in the prophylactic vaccine preparations provides initial protection against tumor development; however, is not effective in providing protection against further tumor challenges.

## Discussion

A challenge that immunotherapies face is to overcome an immunosuppressive TME to enhance an anti-tumor immune response (49, 50). Prophylactic therapies, such as cancer vaccines, promote an anti-tumor immune response in the absence of an immunosuppressive TME (3, 51, 52). Although there are different types of cancer vaccines being studied, those vaccines designed to target multiple TAAs increase the potential for tumor recognition and reduce the potential for escape variants (53–58). Using whole tumor cells provides access to all potential TAAs, and targets that may be unidentified for use by immune cells. In addition, whole tumor cell vaccines that incorporate dead or dying tumor cells resulting from ICD provide a more robust immune response (59, 60), and constitute what we refer to as a DTCV. The robust immune response associated with induction of ICD is attributed to the increase in DAMPs including high HMGB1 (61, 62), HSPs (63, 64), and pentraxin-3 (PTX3) (65), and ATP (66). In addition to the presence of DAMPs, exposure to irradiation can increase the expression of calreticulin (CRT) and phosphatidylserine (PS) on the surface of the cancer cells (67–70) which also contributes to immune recognition of dying cells.

Previous studies have reported that the protocol used for generating a DTCV impacts their efficacy. For example, cancer vaccines consisting of apoptotic cells demonstrated better protection than necrotic cells in colon, melanoma, and renal cancer models (15). However, opposing results have been reported in melanoma where comparable protection was observed when DCs were pre-loaded with necrotic or apoptotic cancer cells (71). Exposing cancer cells to irradiation, UV-irradiation and γ-irradiation can result in apoptosis (72, 73), while necrosis can be achieved through repeated F/T cycles, with the number of cycles contributing to the necrotic state of the cells (74). UV and γ-irradiation have previously been compared in colon cancer (75), and in human melanoma (76). Vandenberk et al. showed that irradiating lysed tumor cells in a model of high-grade glioma is more effective than either irradiation or lysis alone (13). In the present study, we focused on comparing four different protocols used to generate a DTCV, consisting of cancer cells that were either exposed to lysis, UV-irradiation, LyUV, or γ-irradiation. LyUV utilized a single round of F/T as multiple rounds induced more necrosis and debris, while a single cycle had the potential to promote antigen cross-presentation, while also keeping membranes intact (22, 74). By using a single round of F/T, subsequent exposure to UV-irradiation, which is the case in our LyUV treatment, has the potential to act on tumor cells that may have partially intact membranes.

We found that LyUV, although by visual observation appears to be a combination of lysis and UV-irradiation. Further analysis by flow cytometry 24 hrs. post induction of cell death results in minimal differences between lysis, UV-irradiation and LyUV. However, at the 3 hr time point after induction of cell death, there are greater differences seen in the flow cytometry analysis, with LyUV resembling UV-irradiation, although UV-irradiation alone yielded live cells. Lysis and LyUV resulted in more rapid induction of cell death when compared to UV- or γ-irradiation alone. The induction of cell death can increase the potential of TAA acquisition by APCs. With antigen quality being an important factor to consider when designing antigen specific immune responses (77); this may provide insight into the improved protection of LyUV (single round of F/T) compared to lysis (5 rounds of F/T), as repeated F/T cycles may decrease TAA quality (78).

Cancer cells develop immune escape mechanisms (79), and each of these mechanisms highlight challenges and potential targets for cancer immunotherapies. An example of this is through the downregulation of MHC-I on cancer cells, making these cells less immunogenic (80) and unable to be detected by CD8^+^ T cells (24). We show that exposure to both UV- and γ-irradiation can increase MHC-I expression, and in the B16-OVA model, UV-irradiation was able to increase MHC-I expression to a greater extent than γ-irradiation at the doses compared. We were not able to test MHC-I expression levels following exposure to lysis or LyUV because the large increase in cellular debris present after 24 hrs. of incubation rendered these protocols of inducing cell death unsuitable for analysis by flow cytometry. However, with LyUV having the potential to yield intact cells these cells may have increased MHC-I expression that could promote CD8^+^ T cell recognition of the tumor cells. Although the focus of inducing cell death was to evaluate changes in MHC-I expression, which would have the potential to improve CD8^+^ T cell recognition of cancer cells, future studies could also investigate how DTCVs could impact CD4^+^ T cell development, as CD4^+^ T cells can help promote and sustain anti-tumor CD8^+^ T cell responses (81).

Appropriate DC stimulation can promote effective T cell activation, while the absence of appropriate co-stimulation can lead to T cell anergy (82). In our study, supernatants from dead cancer cells influenced DC2.4 activation. We observed an increase in the co-stimulatory molecules CD80, CD86 and CD40 following UV and LyUV protocols. Indicating, that the supernatants from these cells can promote co-stimulatory molecule expression on DCs, which are required for T cell activation. Interestingly we observed an increase in CD80 expression following lysis, while following exposure to γ-irradiation we observed a decrease in expression. This may be attributed to the timing at which CD80 expression was observed, as CD80 is increased later when compared to CD86 (83). To this point, supernatants from cancer cells exposed to γ-irradiation did not result in DC upregulation of any markers tested. This may be a result of γ-irradiation yielding many cells in early apoptosis following the 24 hr incubation, leading to fewer DAMPs being present at the time of collection. Previous research has indicated that apoptotic or necrotic tumor cells can activate DCs, while other studies indicated opposing results (84).

We demonstrated that LyUV can induce rapid cancer cell death and effective activation of DCs. The timing of DC activation and maturation can dictate antigen cross-presentation (20, 85). We found that DC phagocytosis of cancer cells was greater 3 hrs. after LyUV compared to UV-irradiation or γ-irradiation but not that of lysis. If cancer cells were left for 24 hrs. before co-incubation with DCs, LyUV still resulted in greater phagocytosis than UV- or γ-irradiation, but the difference between lysis and LyUV was not significant. This indicates that LyUV can provide the benefits of lysis (increased antigen acquisition) and the benefits of UV-irradiation (DC activation). Furthermore, induction of cell lysis by F/T cycles has been shown to be poor at activating the immune response (86). However, cell lysis results in the formation of cellular fragments that are easier for acquisition by DCs. This may help indicate why a more significant amount of phagocytosis was observed with lysis when compared to LyUV. Moreover, DCs in an immature state are well recognized for their capacity for endocytosis of extracellular components, however upon maturation they have reduced antigen acquisition and improved antigen processing and presentation (87). This may explain why cell lysis would allow a higher degree of phagocytosis while not improving DC activation as compared to LyUV.

We next wanted to determine how other protocols for generating DTCVs influenced protection against tumor challenge. A prime-boost model was used because it provides better protection than a single vaccination in our model. We found that LyUV, but not lysis or UV-irradiation, significantly improved protection compared to PBS. B16-OVA cells exposed to UV-irradiation and γ-irradiation had comparable effects on DC activation and resulting phagocytosis, however γ-irradiation provided better protection against tumor challenge than UV-irradiation. This may be attributed to the high dose of UV irradiation used in this study impacting the resulting cell death observed following injection. In contrast γ-irradiation results in a more gradual induction of cell death going through early apoptotic and late apoptotic stages. However, at the high dose of UV-irradiation used here the cells progress from live to dead rapidly after vaccination, potentially explaining the reduced efficacy in protection observed. Although others have reported that cancer cell death induced by lysis or UV-irradiation alone or antigen preloaded DCs can enhance protection (71, 88). The increase in the protection provided by LyUV was comparable to γ-irradiation. These results indicate that LyUV can be used as an additional protocol of inducing cell death that can be completed faster and safely.

Studies have explored using irradiated tumor cells that are transduced with genes encoding an adjuvant, such as a cytokine, in cancer vaccines to help orchestrate a desired immune response (89, 90). With LyUV, it is not possible to transfect these cells and ensure continual expression of the desired cytokine after exposure. Therefore, with incomplete protection and an inability to transfect LyUV exposed B16-OVA cells, we next wanted to determine how the addition of a recombinant cytokine could improve the efficacy of our DTCVs.

IL-12 has been explored as a cancer vaccine adjuvant by promoting anti-tumor effects (30, 91), however, the use of IL-12 has been limited due to toxicity concerns (92, 93). Studies have used inducible IL-12 expression in chimeric antigen receptor (CAR) T cell therapy to reduce toxicity associated with IL-12 (94). Furthermore, IL-27 is a member of the IL-12 family of cytokines and is well tolerated and does not exhibit toxicity concerns (31). In the current study, we asked whether rmIL-27 would be capable of enhancing the efficacy of the DTCV. IL-27 can promote Th1 differentiation (95, 96), while also providing enhanced CTL activation *in vivo* (97, 98). In addition, IL-27 can improve DC-mediated antigen presentation and result in Treg depletion (35, 99). However, IL-27 has been identified as a pleiotropic cytokine (32) with the capacity to also activate Tregs (100) and induce immunosuppression through DCs (101, 102), which may depend on their maturation state (103). In this study, we tested two different doses of rmIL-27 to determine the effects of IL-27 in our DTCVs. We observed that rmIL-27 was able to enhance protection by both LyUV and γ-irradiated DTCVs; however, the dose of rmIL-27 drastically impacted the efficacy of the vaccine in the B16-OVA model used. Interestingly, the addition of rmIL-27 at a lower dose (10 ng/mouse) to vaccinations consisting of LyUV or γ-irradiated B16-OVA cells provided significant increases in survival of mice challenged with live tumors. However, at a higher concentration of rmIL-27 (100ng/mouse), there was a decrease in protection compared to LyUV or γ-irradiated cells alone. This indicates the potential of rmIL-27 at higher doses having an immunosuppressive effect. Previous studies using IL-27 transfected B16 cells have non-specified concentrations (104, 105), while delivery using viral vectors yields varying concentrations (35, 106). The levels of IL-27 may be an important factor to consider where higher amounts may reduce the potential for an anti-tumor immune response to ensue in a prophylactic model.

With a significant improvement in survival observed with the addition of rmIL-27 at a lower dose, we asked if the surviving mice could be protected against tumor rechallenge. Regardless of the protocol used to induce cell death, or the addition of rmIL-27 to the DTCVs, mice that originally demonstrated protection did not survive longer than 30 days following tumor rechallenge. This may indicate that in our model, rmIL-27 can promote anti-tumor effector T cell responses without the induction of memory. Other researchers have indicated that IL-27 is required for the induction of T cell activation and memory in response to immunization (107, 108). The development of effector versus memory responses can be dictated by distinct cytokines and transcription factors (109). Based on our results, it appears that the effects of IL-27 as an adjuvant in a DTCV to develop anti-tumor responses are not long-lasting and could be due to the lack of establishing robust memory responses. The lack of a robust memory response could also be attributed to weak induction of CD4^+^ T cell help, which is required for memory CD8^+^ T cell generation (81). However, the presence of IL-27 during the initial vaccination may help promote innate immune cell activation to establish strong anti-tumor responses. NK cells can also respond to IL-27 and promote effector cell function in viral and tumor models (110, 111). Therefore, future research should investigate how a prophylactic vaccine consisting of tumor cells exposed to a method of ICD and IL-27 affects T cell (effector and memory) and NK cell responses.

Although LyUV and γ-irradiation could protect against tumor challenges, it is essential to recognize the limitations of these methods. The main restriction is the need to obtain high cell number; unlike DNA and peptide cancer vaccines, where the target can be synthetically made, DTCVs require isolating tumor cells in large quantities. The DTCVs evaluated here would be easier to prepare and deliver following tumor resection from the patient, to prevent tumor recurrence, similarly to that of BCG vaccination protocol to avoid the recurrence of bladder cancer (112). These challenges would not be present if a DTCV was used therapeutically, where the tumor cells would be detectable and accessible. IL-27 is known to directly impact both CD4^+^ and CD8^+^ T cells and both cell types have a role in promoting cancer vaccine efficacy. Therefore, future studies should investigate the mechanisms involved in establishing the increase in DTCV efficacy with the addition of IL-27.

In conclusion, we demonstrate in a prophylactic setting that different protocols to induce cell death can impact the efficacy of DTCVs and that the LyUV is a robust protocol of inducing cell death that can give significant protection against tumor growth *in vivo.* Moreover, we highlight the potential of using rmIL-27 as an adjuvant to improve cancer vaccines, while emphasizing the importance of dose consideration. Therefore, IL-27 shows promise in enhancing anti-tumor responses but requires further investigation into the mechanisms that are responsible for its pleiotropic effects.

## Data Availability Statement

The original contributions presented in the study are included in the article/**Supplementary Material**. Further inquiries can be directed to the corresponding author.

## Ethics Statement

The animal study was reviewed and approved by Queen’s University Animal Care Services.

## Author Contributions

KS, KG, and SB conceptualized the project. KS and OK performed experiments. KS analyzed and wrote the manuscript. PG provided experimental insight. All authors reviewed and edited the manuscript.

## Conflict of Interest

The authors declare that the research was conducted in the absence of any commercial or financial relationships that could be construed as a potential conflict of interest.

## Publisher’s Note

All claims expressed in this article are solely those of the authors and do not necessarily represent those of their affiliated organizations, or those of the publisher, the editors and the reviewers. Any product that may be evaluated in this article, or claim that may be made by its manufacturer, is not guaranteed or endorsed by the publisher.

## Supplementary Material

Supplementary Figure 1 Evaluation of propidium iodide staining following exposure to different numbers of F/T cycles. Cancer cells were exposed to the indicated number of rounds of F/T cycles (0, 1, or 5) and subsequently stained with propidium iodide (1.5 μg/mL) and analyzed *via* flow cytometry. **(A)** Fold change in MFI compared to untreated cells with data expressed as ± SD of three independent experiments. **(B)** Representative dot plot of total PI positive cells as a percent following the indicated number of rounds of F/T cycles. The percent PI positive cells are shown in the dot plots. n = 3, ***p* < 0.01. ns denotes not significant.

Supplementary Figure 2 Comparison of different doses of γ-irradiation on B16-OVA cell death 24 hrs. after treatment. Cancer cells were exposed to the indicated dose of γ-irradiation and left to incubate for 24 hrs. Cells were then harvested, and the type of cell death was determined through AV/PI staining and flow cytometry.

Supplementary Figure 3 Prophylactic cancer vaccine consisting of γ-irradiated B16-OVA cells delivered in a prime-boost vaccination model provides better protection than a single vaccination with DTCV. γ-irradiated B16-OVA cancer cells were injected intraperitoneally (i.p.) at 5.0x10^6^ cells/mouse: either once, one week before engraftment; or twice, one and two weeks before engraftment. Seven days after the final vaccination, mice were engrafted with 1.0x10^6^ live B16-OVA cells subcutaneously and tumour growth was monitored. Kapan-Meier survival analysis, n = at least 5. **p* < 0.05, ns denotes not significant.

Supplementary Figure 4 The addition of IL-27 at a lower dose to the prophylactic cancer vaccine consisting of LyUV-treated B16 cells improved protection. B16 cells (not expressing OVA) were exposed to LyUV treatment and were injected intraperitoneally (i.p.) at 5.0x10^6^ cells/mouse in the absence or presence of rmIL-27 at 10ng/mouse or 100 ng/mouse. The vaccine was delivered 14 and 7 days before tumour engraftment with 1.0x10^6^ B16 cells injected subcutaneously on day 0. Tumour outgrowth was then monitored. Kapan-Meier survival analysis, n = 3. **p* < 0.05.
