# Supplementary figures and images for: IL-27 Improves Prophylactic Protection Provided by a Dead Tumor Cell Vaccine in a Mouse Melanoma Model
Source: Front Immunol. 2022 Apr 21;13:884827. doi: 10.3389/fimmu.2022.884827 (PMC9069009; doi:10.3389/fimmu.2022.884827)

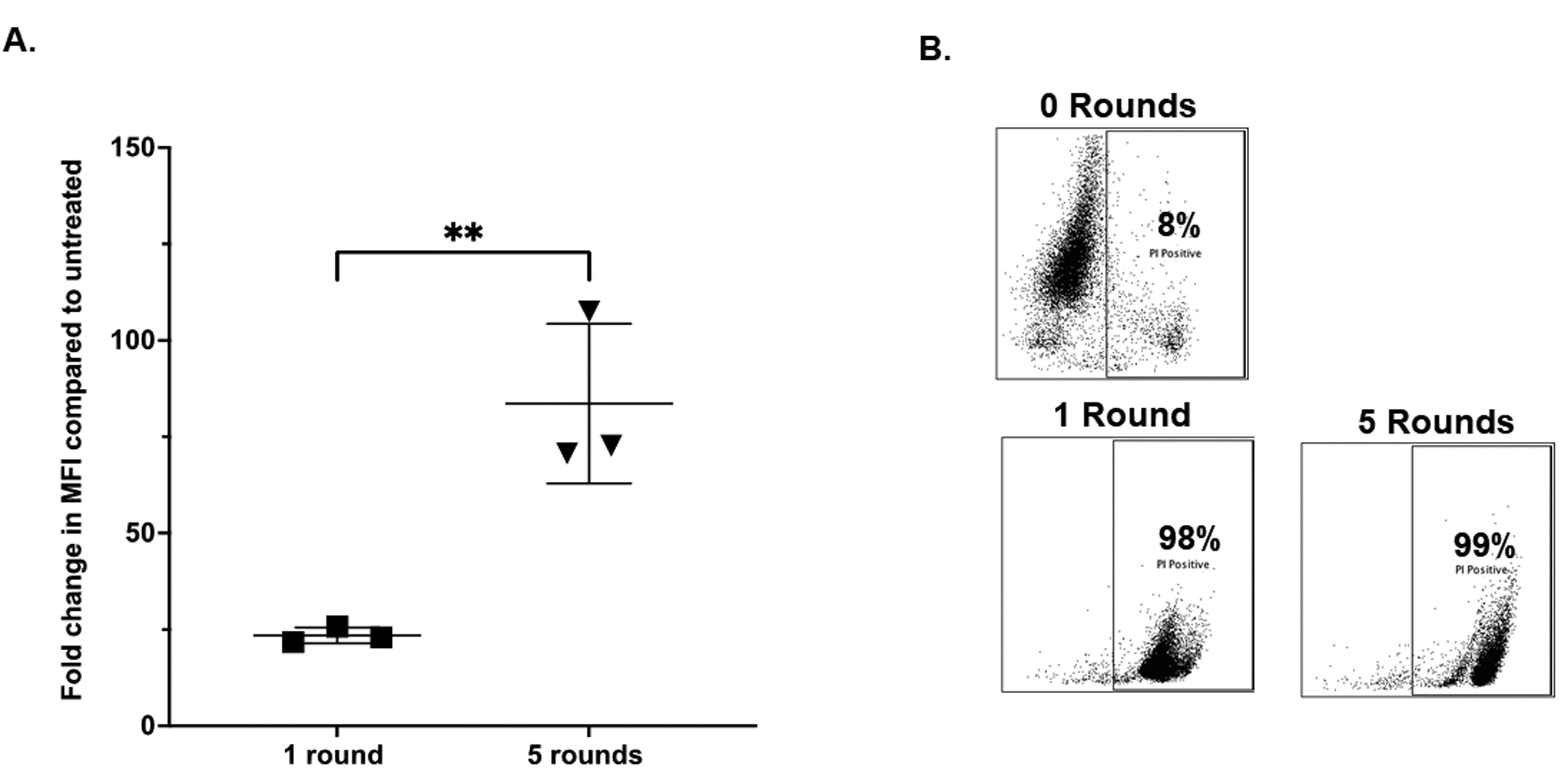

Supplement: Supplementary Figure 1 — Evaluation of propidium iodide staining following exposure to different numbers of F/T cycles. Cancer cells were exposed to the indicated number of rounds of F/T cycles (0, 1, or 5) and subsequently stained with propidium iodide (1.5 μg/mL) and analyzed via flow cytometry. (A) Fold change in MFI compared to untreated cells with data expressed as ± SD of three independent experiments. (B) Representative dot plot of total PI positive cells as a percent following the indicated number of rounds of F/T cycles. The percent PI positive cells are shown in the dot plots. n = 3, **p < 0.01. ns denotes not significant. [file Image_1.tif]

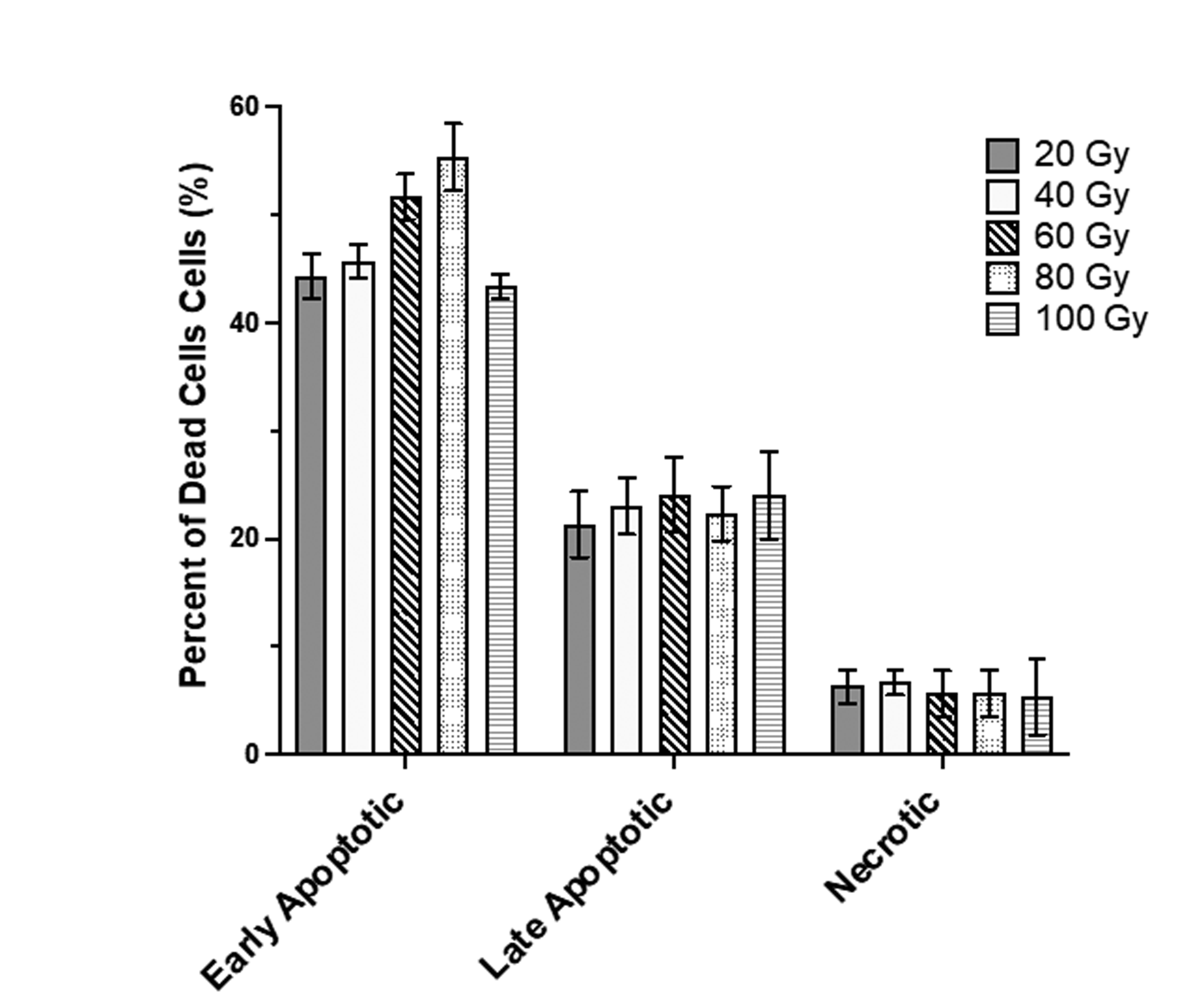

Supplement: Supplementary Figure 2 — Comparison of different doses of γ-irradiation on B16-OVA cell death 24 hrs. after treatment. Cancer cells were exposed to the indicated dose of γ-irradiation and left to incubate for 24 hrs. Cells were then harvested, and the type of cell death was determined through AV/PI staining and flow cytometry. [file Image_2.tif]

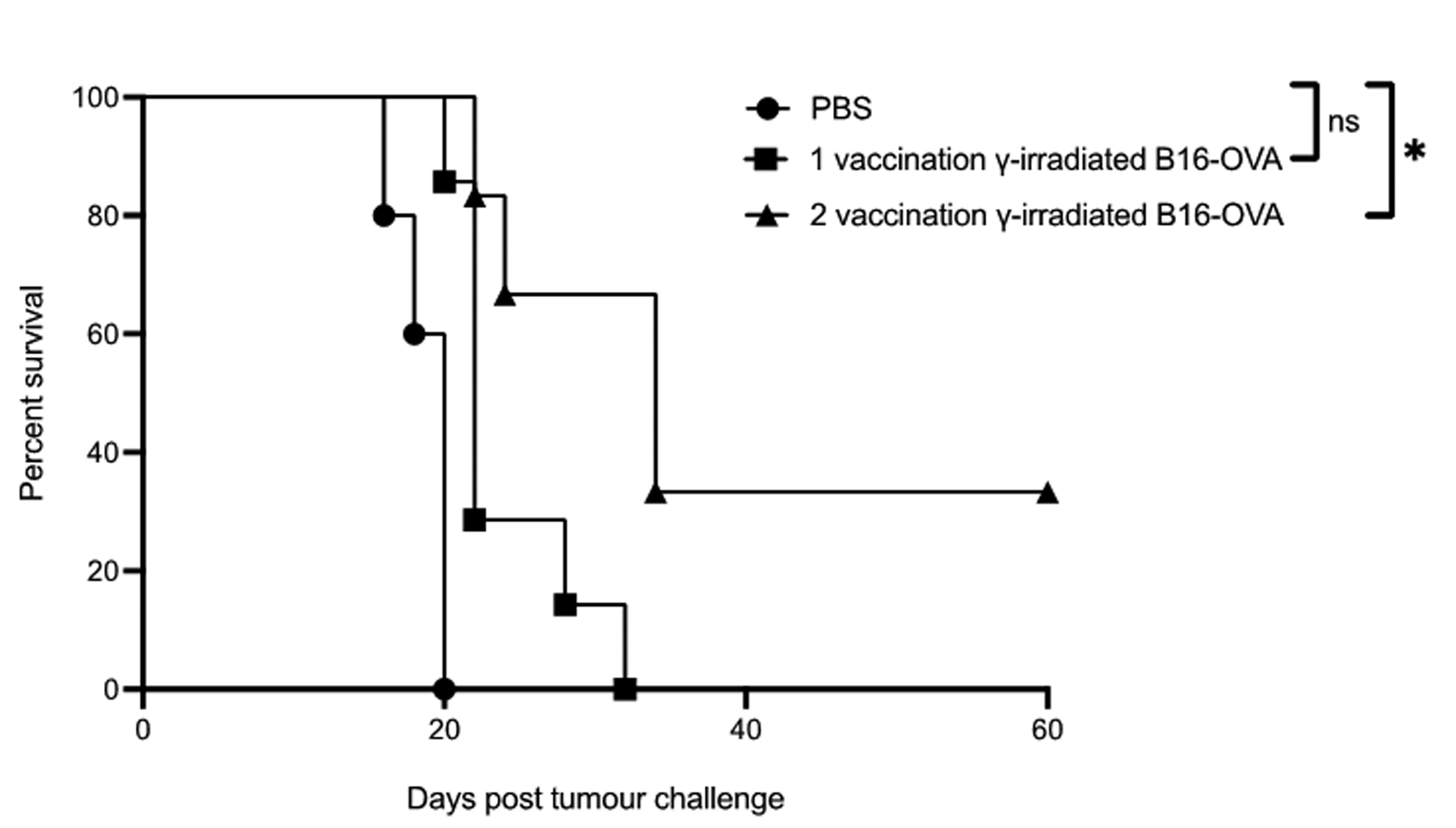

Supplement: Supplementary Figure 3 — Prophylactic cancer vaccine consisting of γ-irradiated B16-OVA cells delivered in a prime-boost vaccination model provides better protection than a single vaccination with DTCV. γ-irradiated B16-OVA cancer cells were injected intraperitoneally (i.p.) at 5.0x10^6^ cells/mouse: either once, one week before engraftment; or twice, one and two weeks before engraftment. Seven days after the final vaccination, mice were engrafted with 1.0x10^6^ live B16-OVA cells subcutaneously and tumour growth was monitored. Kapan-Meier survival analysis, n = at least 5. *p < 0.05, ns denotes not significant. [file Image_3.tif]

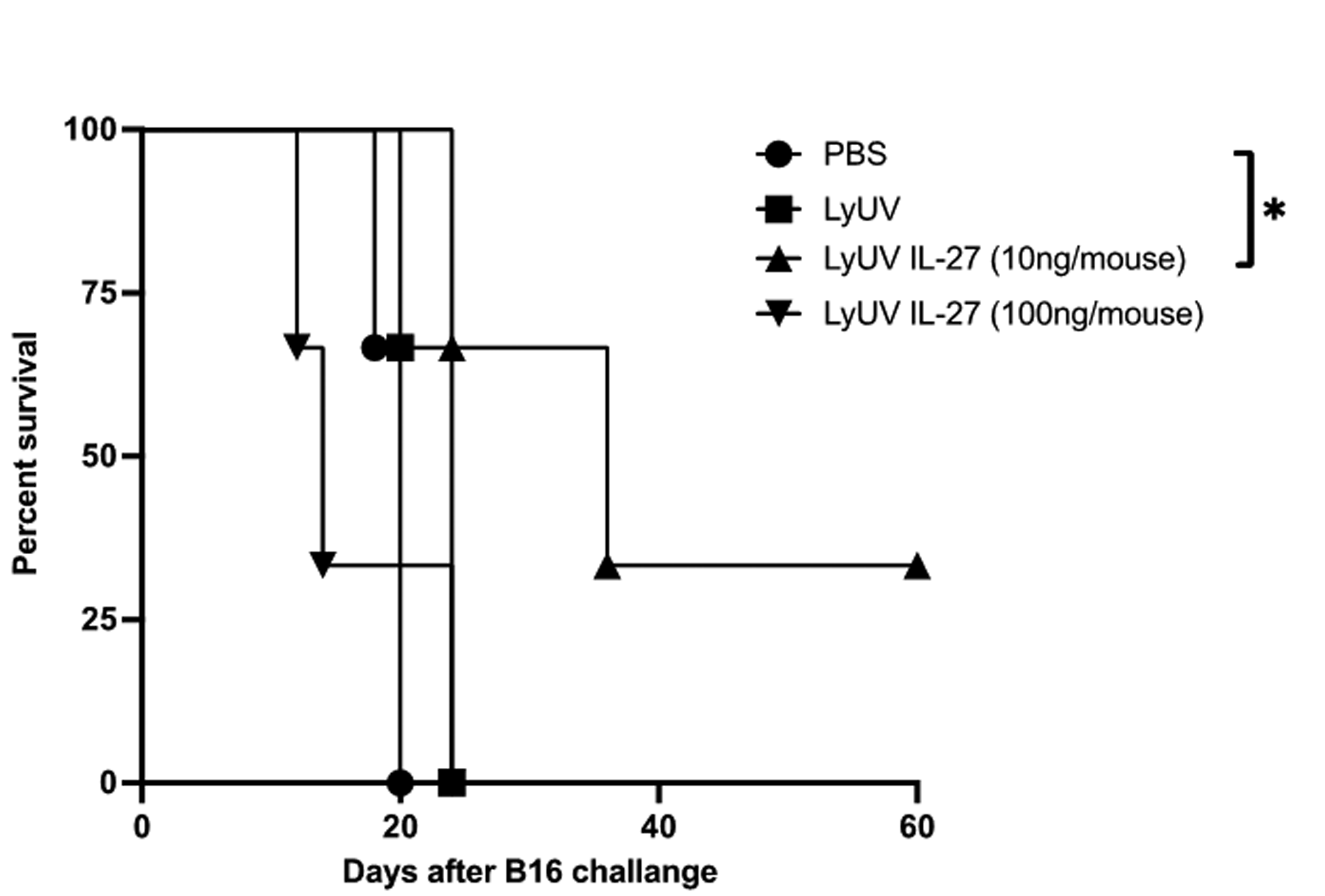

Supplement: Supplementary Figure 4 — The addition of IL-27 at a lower dose to the prophylactic cancer vaccine consisting of LyUV-treated B16 cells improved protection. B16 cells (not expressing OVA) were exposed to LyUV treatment and were injected intraperitoneally (i.p.) at 5.0x10^6^ cells/mouse in the absence or presence of rmIL-27 at 10ng/mouse or 100 ng/mouse. The vaccine was delivered 14 and 7 days before tumour engraftment with 1.0x10^6^ B16 cells injected subcutaneously on day 0. Tumour outgrowth was then monitored. Kapan-Meier survival analysis, n = 3. *p < 0.05. [file Image_4.tif]
